# Evaluation of the incidence of obstructive sleep apnea in mandibular fracture patients before and after treatment with the STOP-BANG questionnaire

**DOI:** 10.4317/medoral.27047

**Published:** 2025-08-16

**Authors:** Mahdi Jafari, Sahand Samieirad, Rozhin Kafshdar Goharian, Ricardo Grillo

**Affiliations:** 1 Dentistry Student, Faculty of Dentistry, Mashhad University of Medical Sciences, Mashhad, Iran; 2 Professor, Faculty of Dentistry, Mashhad University of Medical Sciences, Mashhad, Iran; 3 Professor, Department of Oral and Maxillofacial Surgery, University of São Paulo, São Paulo, Brazil

## Abstract

**Background:**

Mandibular fractures are common injuries, leading to various complications, including obstructive sleep apnea (OSA). The aim of this study was to evaluate the occurrence of OSA in relation to mandibular fracture type before and after treatment using the STOP-BANG questionnaire.

**Material and Methods:**

This prospective study was conducted on patients admitted to Shahid Kamyab Hospital from 2022 to 2023 with mandibular fractures. Patients were classified based on the type and location of fracture, age, gender, and cause. The primary predictor variable was the type of mandibular fracture. The primary outcome variable was the occurrence of OSA as measured by the STOP-BANG questionnaire. Secondary outcomes included changes in STOP-BANG scores over time before and after treatment. Covariates were divided into age and gender (demographic), type of surgical treatment, and Body Mass Index (physiologic). Data analyses included comparisons of STOP-BANG scores over multiple time points (pre-fracture [T0], post-fracture [T1], 1 week post-surgery [T2], and 1 month post-surgery [T3]). The results were analyzed using SPSS 16 software, with a significance level set at *p-value* < 0.05.

**Results:**

In this study, 154 patients were examined. Ninety-five patients had unilateral fractures and 59 patients had bilateral fractures. The study groups were homogeneous in terms of age, gender, surgical method, and Body Mass Index (BMI). In both unilateral and bilateral groups, the average STOP-BANG score changed significantly over time (*p*<0.001). Pairwise comparisons indicated that the STOP-BANG score significantly increased at all times compared to pre-fracture, but decreased significantly at each subsequent time point after the fracture. The mean STOP-BANG score was significantly higher in the bilateral group compared to the unilateral group at each time point (T1, T2, T3) (*p*<0.001). The mean changes in STOP-BANG score relative to T0 were significantly greater in the bilateral group compared to the unilateral group (*p*<0.001).

**Conclusions:**

Mandibular fractures affect respiratory conditions and can lead to OSA. Surgeons should consider the reduction in respiratory space when treating these patients and choose an appropriate treatment plan. Bilateral fractures are more likely to lead to OSA, whereas OSA occurrence was rarely observed in cases of unilateral fractures.

** Key words:**Obstructive sleep apnea, mandible, maxillofacial injuries, surveys and questionnaires, postoperative complication.

## Introduction

Jaw and facial fractures are prevalent injuries with varying causes. In developed countries, these fractures commonly result from road accidents, while in developing nations, they often occur due to conflicts. Other frequent causes include military-related trauma, falls in children, and sports injuries. Mandibular fractures, in particular, constitute a significant portion of these injuries and can lead to complications such as malocclusion, malnutrition, and respiratory problems, making their treatment crucial (1,2). Mandibular fractures are classified primarily based on fracture location—condylar, angular, body, symphysis, and parasymphysis fractures. These fractures may also present in combinations, complicating classification and treatment (3). The close proximity of the mandible to vital structures such as blood vessels, nerves, and muscles, as well as potential disruption to dental occlusion, underscores the importance of effective treatment (4-7).

Treatment methods vary from conservative approaches like intermaxillary fixation and occlusal splints to more invasive techniques such as open reduction and internal fixation (6). In the closed method, the mandible and maxilla are fixed with an arch bar, allowing natural healing. The open method involves surgical exposure and fixation with screws and plates. Treatment decisions depend on fracture displacement, location, and occlusal disruption (8). A noTable complication of mandibular fractures is respiratory tract disturbance. Fractures or treatment-related displacement can alter the respiratory space due to muscle repositioning (7,9). Obstructive sleep apnea (OSA), characterized by intermittent airway obstruction during sleep, affects 3-7% of the population and is associated with a range of health issues, including cardiovascular diseases and metabolic disorders (10-14). OSA is multifactorial, influenced by anatomical abnormalities and surgical interventions (15-24). Accurate diagnosis is crucial to prevent related complications. While polysomnography (PSG) is the gold standard for diagnosing OSA, it is expensive and not always feasible for large-scale screening (9,14,25). Consequently, alternative screening methods, such as the STOP-BANG questionnaire, have been developed and validated (14,26-28).

Given the correlation between jaw fractures and airway changes, and the limitations of PSG, this study aims to investigate the incidence of OSA following mandibular fractures using the STOP-BANG questionnaire. The specific aims are: 1) to measure the incidence of OSA in patients with mandibular fractures; 2) to compare the incidence based on the type of fracture; and 3) to assess the relationship between fracture type and OSA occurrence.

## Material and Methods

This study was performed in the period of March 2022 to March 2023 at the location of Velayat- Hospital and Madar Hospital. The STOP-BANG questionnaire was used in this study. A double-blinded experimental study was carried out. This was a before-after study, and its protocol was approved by the Ethics and Research Committee of Mashhad University of Medical Sciences. After obtaining written informed consent, all healthy (American Society of Anesthesiologists Classification I and II) patients who had referred to Velayat Hospital and Mader Hospital due to mandibular fracture were classified according to the type and location of fracture, age, cause, and gender, and were included in the study.

Inclusion Criteria were: 1) Mandibular fracture patients referred to Velayat and Mader Hospital; 2) No history of previous maxillofacial surgeries; 3) No history of respiratory diseases during sleep; 4) Patients with systemic ASA 1,2. Exclusion criteria were: 1) Patients whose treatment and surgery were accompanied by unforeseen problems; 2) Patients who lost follow-up; 3) Patients who did not want to cooperate in the study plan. No intervention was performed in this study and therefore there was no need for a control group.

- Indicators/variables including exposure, outcome, confounders and how they are measured

The main variable of this study is the type of mandibular fracture and the treatment method. The dependent variable in this study is the incidence and rate of obstructive sleep apnea. Also, age, gender, BMI and neck circumference were considered as background variables. Respiratory diseases such as rhinitis, bronchitis, asthma, etc. were considered as confounding variables in this study and were excluded.

- Main measurable outcomes

OSA which is measured in 4 intervals using the STOP-BANG questionnaire. T0, which is based on the conditions before the fracture, T1 immediately after the occurrence of the fracture and before treatment, T2 one week after the mandibular fracture treatment and T3 interval 1 month after the treatment.

- Relationship between obstructive sleep apnea and different types of mandibular fractures before and after treatment

All patients were asked to complete 2 STOP-BANG questionnaires before surgery, once based on pre-fracture conditions (T0) and once based on post-fracture conditions (T1). All the information obtained from the questionnaires were recorded in the checklist for each patient. Also, in this checklist, other information such as age, gender, type and location of fracture and details of the patient's surgery were mentioned.

This questionnaire is designed in 2 parts, the questions of the first part include snoring (s), daily fatigue (Tiredness during daytime (D)), observed apnea (O) and high Blood pressure (High blood pressure (P)), (STOP); And the questions of the second part include BMI (B), age (A), neck circumference (N) and gender (G), (BANG). In other words, this questionnaire includes 8 questions about continuous loud snoring in sleep, daily fatigue, breathing interruption during sleep, history of high blood pressure, body mass index over 35, age over 50 years, neck circumference over 40 cm and Gender is male, which screens the possibility of obstructive sleep apnea in the form of yes (1) and no (0). After the treatment, the patients were asked to complete 2 more STOP-BANG questionnaires in the intervals of one week after treatment (T2) and one month after treatment (T3).

- Statistical methods and sample size

Based on past experiences, the sample size was 100 people. Appropriate statistical Tables and graphs were used to describe the data, using SPSS® v 16.0 (IBM, USA). Analysis of variance with repeated measures and Cochran's was used to analyze the data. Also, the level of significance in the test was considered equal to 5%.

## Results

The present study included 154 people including 33 women (21.4%) and 121 men (78.6%) with an average age of 30.97 ± 8.74 years and an age range of 12 to 52 years. Patients were classified based on BMI, type of fracture (unilateral or bilateral), location of fracture, and type of treatment, and were evaluated for OSA score using the STOP-BANG questionnaire (SBQ) at 4-time intervals (T0: before fracture, T1: after fracture and before treatment, T2: one week after treatment, T3: one month after treatment).

**Figure 1:**
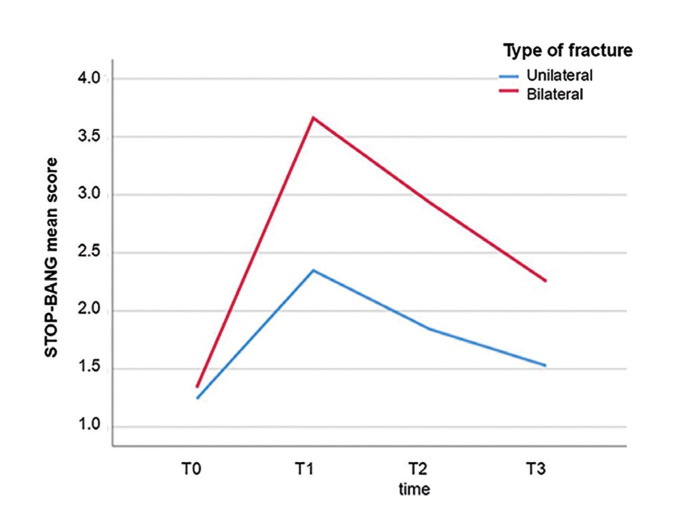
Mean SBQ score during the studied times by groups.

The mean score of SBQ in the bilateral group was significantly higher than the unilateral group in all time intervals except T0. Out of a total of 154 patients, 95 (61.7%) presented with unilateral fractures and 59 (38.3%) presented with bilateral fractures. 85 patients (55.2%) were treated with the Close method and 69 patients (44.8%) with the Open method (Fig. 1).

**Figure 2:**
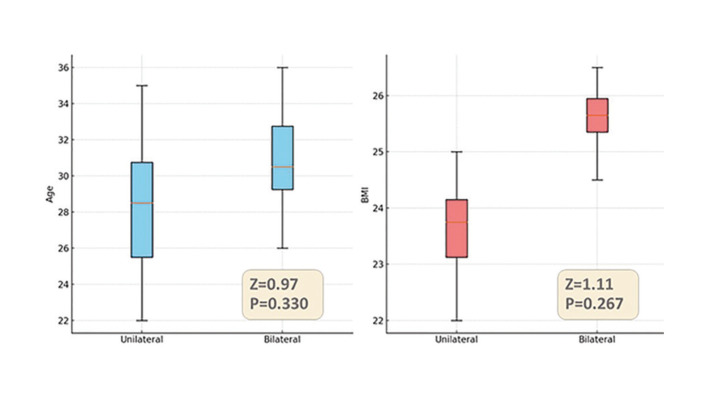
Comparison between age (blue) and BMI (red) by groups.

The number, average, standard deviation, minimum and maximum variables of age and BMI by groups and the result of the statistical test is shown in Fig. 2. It was found that age and BMI have a direct but non-significant relationship with SBQ scores in both groups (unilateral and bilateral).

In Table 1, no significant differences were found in gender distribution or treatment methods across groups, indicating that the groups were homogeneous in terms of age, gender, surgical method, and BMI. Table 2 shows that in both unilateral and bilateral groups, the SBQ scores increased significantly after the fracture (T1) and then decreased following treatment (T2, T3), though the decrease was not significant from T2 to T3 in the unilateral group. Table 3 highlights that SBQ scores were significantly higher in the bilateral group compared to the unilateral group at T1, T2, and T3. Table 4 reveals that SBQ score changes were significantly greater in the bilateral group compared to the unilateral group at most intervals. Supplement 1 and Supplement 2 show that SBQ score changes varied significantly among fracture sites within both the unilateral and bilateral groups, with specific sites showing greater changes than others at different time intervals. Pairwise comparisons of fracture sites revealed the following results:

1. The mean SBQ changes at T2 relative to T0 were significantly higher in the condyle-condyle site compared to parasymphysis-subcondyle and body-angle sites. No significant differences were observed between other sites.

2. The mean SBQ changes at T3 relative to T0 were significantly lower in the (parasymphysis+condyle) site compared to (parasymphysis+parasymphysis), (condyle+condyle), and (angle+angle) sites. No significant differences were observed between other sites.

## Discussion

The results of this study show a statistically significant increase in SBQ scores during post-fracture periods (T1/T2/T3) compared to pre-fracture baseline (T0). Furthermore, the data indicate a noTable decrease in questionnaire scores during post-treatment periods (T2/T3) relative to the pre-treatment phase (T1), with scores progressively returning to pre-fracture (T0) levels.

Pre-fracture (T0) mean SBQ scores for obstructive sleep apnea were 1.28 ± 0.78 (range: 0-3). These scores increased significantly immediately post-fracture (T1) to 2.85 ± 1.27 (range: 1-6). Subsequently, at one-week post-treatment (T2), scores decreased to 2.26 ± 1.16 (range: 0-6), and at one-month post-treatment (T3), further decreased to 1.81 ± 0.93 (range: 0-5), approaching pre-fracture levels. These mean differences were statistically significant (*p* <0.001).

Statistical analysis revealed significant temporal changes in mean STOP-BANG scores for both unilateral and bilateral fracture groups (*p*<0.001).

No significant difference was observed between unilateral and bilateral groups in STOP-BANG scores at T0 (pre-fracture) (*p*=0.419). However, significantly higher STOP-BANG scores were observed in the bilateral group compared to the unilateral group across all post-fracture time points (T1/T2/T3) (*p*<0.001 for each period).

The primary objective of this study was to investigate changes in STOP-BANG scores before and after mandibular fractures and subsequent treatment in patients, rather than establishing a potential causal relationship between mandibular fractures and the onset of OSA, as the STOP-BANG questionnaire is primarily a screening tool rather than a definitive diagnostic test for OSA.

Maxillofacial fractures show high epidemiological prevalence. In developed nations, these traumas are predominantly attributed to road traffic accidents, while in developing countries, interpersonal violence is the primary cause. Additional etiologies include war-related injuries in military personnel, falls in pediatric populations, and sports-related injuries (1,2).

A significant complication of maxillofacial fractures is the disruption of respiratory pathways (16-18). Mandibular displacement during fracture or treatment can lead to the displacement of attached musculature, potentially resulting in alterations to respiratory space volume and, in severe cases, the onset of obstructive sleep apnea (7,9,21-24).

OSA is a chronic condition characterized by recurrent episodes of upper airway collapse during sleep. OSA is the most prevalent sleep-related breathing disorder, with an estimated prevalence of 3-7%, defined by an Apnea-Hypopnea Index (AHI) ≥ 5 (10,12,13).

Common symptomatology includes persistent snoring, excessive daytime somnolence, observable apneic episodes, fatigue, and hypoxia. Associated comorbidities encompass hypertension, cardiovascular diseases, cerebrovascular accidents, and glucose metabolism abnormalities (14). Upper airway collapse primarily occurs during REM sleep, affecting the oropharynx, velopharynx, and nasopharynx (17,19,21,22). Key predictive factors for OSA include increased neck circumference, tobacco use, mandibular retrognathia, macroglossia, uvular hypertrophy, obesity, advanced age, and racial predisposition.

A meta-analysis by Neelapu *et al*. provided robust evidence of reduced pharyngeal airway space, inferior hyoid bone position, and increased anterior facial height in adult OSA patients compared to controls (25).

Our study’s primary objective was to investigate OSA incidence in patients with mandibular fractures. Consistent with El-Anwar *et al*., our findings indicate that bilateral fractures are associated with a higher incidence of OSA (26). A secondary objective was to examine the relationship between various mandibular fracture types and OSA occurrence. Our results, corroborating previous studies, demonstrate that bilateral mandibular fractures have a higher propensity for OSA development compared to unilateral fractures (26).

While PSG remains the gold standard for OSA diagnosis (14,23), its time-intensive nature, high cost, and limited accessibility render it suboptimal for screening purposes (10,11,14,22,23). Consequently, questionnaire-based screening tools have been developed (14,27).

Among these, the Berlin questionnaire, STOP-BANG questionnaire, and Epworth Sleepiness Scale have demonstrated high sensitivity and specificity in recent studies, particularly in Iranian populations (14,28,29).

Given the limitations associated with PSG and rhinomanometry, including restrictions on sample size (10,11,14,23), we opted to employ the STOP-BANG questionnaire for OSA screening in patients with mandibular fractures. The validity and reliability of this instrument have been established in both domestic and international studies for OSA detection in high-risk populations (14).

While our study shares similarities with El-Anwar *et al*., we utilized the STOP-BANG questionnaire instead of PSG, allowing for a larger sample size and more frequent patient evaluations (26). A noTable advantage of our methodology was the ability to retrospectively assess pre-fracture conditions using the STOP-BANG questionnaire, a limitation in studies relying solely on PSG (26).

Our study is distinguished by its multiple time-point evaluations of respiratory conditions (26). The inclusion of a one-week post-operative assessment (T2) allowed for the evaluation of intermaxillary fixation (IMF) effects on STOP-BANG scores and, by extension, respiratory function. Our findings, consistent with previous studies, indicate that IMF can adversely affect respiratory function and increase STOP-BANG scores.

To our knowledge, this is the first study to utilize the STOP-BANG questionnaire in evaluating the relationship between mandibular fractures and OSA occurrence. Previous investigations have employed alternative methods such as rhinomanometry and polysomnography (PSG) (26).

Our findings suggest that bilateral mandibular fractures are associated with an increased risk of OSA, which can be mitigated through appropriate treatment selection. Surgeons should be cognizant of potential reductions in respiratory space following mandibular fractures and incorporate this consideration into treatment planning. These results align with those reported by El-Anwar *et al* (26).

The results of this study show a significant increase in STOP-BANG questionnaire (SBQ) scores following mandibular fractures, with scores peaking immediately after the injury and gradually decreasing after treatment, returning close to pre-fracture levels. The primary objective was to assess changes in SBQ scores before and after injury and treatment, highlighting the impact of trauma and subsequent swelling on airway function. As the swelling subsides over time, the SBQ scores tend to revert to baseline, underscoring the transient nature of these changes. This study's use of the STOP-BANG questionnaire as a screening tool, despite not being a definitive diagnostic test for OSA, provided valuable insights into the effects of mandibular fractures on respiratory function.

Despite the validated accuracy of the STOP-BANG questionnaire, PSG remains the gold standard for OSA diagnosis. Financial constraints precluded the use of PSG and ApneaLink devices in our study, a limitation that should be acknowledged. The absence of long-term (one-year) follow-up data, due to patient non-compliance, represents another limitation of this investigation. Future studies should consider incorporating PSG and ApneaLink devices for OSA evaluation. Extended follow-up periods (e.g., one year) are recommended to assess long-term outcomes.

Using the findings from this study, one could hypothesize that mandibular fractures exacerbate pre-existing obstructive sleep apnea (OSA) and that the type of fracture (unilateral versus bilateral) and timing of surgical intervention significantly influence the progression of OSA symptoms, as measured by the STOP-BANG questionnaire. A future study could explore this hypothesis by including patients with diagnosed OSA who sustain mandibular fractures, subdividing them based on fracture type and timing of surgery. By assessing changes in STOP-BANG scores at multiple time points, the study could determine whether early surgical intervention is more effective in managing OSA symptoms. While the STOP-BANG questionnaire provides valuable screening insights, the study's limitations, including the absence of polysomnography (PSG) due to financial constraints and a lack of long-term follow-up data, should be addressed in future research. Incorporating PSG and extending follow-up periods could provide a more comprehensive understanding of the impact of mandibular fractures on OSA and help refine surgical decision-making for this patient population.

## Conclusions

While no significant difference in STOP-BANG scores was observed between unilateral and bilateral fracture groups pre-fracture (T0; *p*=0.419), significantly higher scores were noted in the bilateral group across all post-fracture time points (T1/T2/T3; *p*<0.001). Our findings indicate that mandibular fractures can adversely affect respiratory function and potentially precipitate OSA. Bilateral fractures appear to confer a higher risk of OSA development compared to unilateral fractures. These results underscore the importance of considering potential respiratory space reduction in treatment planning for mandibular fractures. The STOP-BANG questionnaire represents a valuable tool for OSA risk assessment in this patient population, facilitating appropriate precautionary measures.

## Figures and Tables

**Table 1:** Comparison of gender and treatment methods between the groups.

Variable		Group			p value
		Unilateral	Bilateral	Total	
Gender	Female	21(22.1%)	12(20.3%)	33(21.4%)	χ2=0.07 p=0.795
	Male	74(77.9%)	47(79.7%)	121(78.6%)	
Treatment method	Open	38(40%)	31(52.5%)	69(44.8%)	χ2=2.31 p=0.128
	Close	57(60%)	28(47.5%)	85(55.2%)	

X^2^, Chi-squared test.

**Table 2:** Comparison of SBQ scores between time intervals by group.

Group	Time	N	Mean ± SD	Interquartile range	Min	Max		p value
Unilateral	T0	95	1.24±0.78	1.0(1.0)	0.0	3.0	1.78^a^	χ2=159.4 p<0.001
	T1	95	2.35±1.00	2.0(1.0)	1.0	6.0	3.43^b^	
	T2	95	1.84±0.88	2.0(1.0)	0.0	5.0	2.64^c^	
	T3	95	1.53±0.73	1.0(1.0)	0.0	3.0	2.15^ac^	
Bilateral	T0	59	1.34±0.78	1.0(1.0)	0.0	3.0	1.25^a^	χ2=142.2 p<0.001
	T1	59	3.66±1.24	3.0(2.0)	2.0	6.0	3.70^b^	
	T2	59	2.93±1.24	3.0(2.0)	1.0	6.0	2.88^c^	
	T3	59	2.25±1.04	2.0(1.0)	0.0	5.0	2.16^d^	

T0, before fracture; T1, after fracture and before treatment; T2, 1 week after treatment; T3, 1 month after treatment. X2, Friedman test. *: Similar lowercase letters indicate no significant difference between tenses.

**Table 3:** Comparison of STOP BANG scores between groups by time intervals.

Time	Group	N	Mean ± SD	Interquartile range	Min	Max	p value
T0	Unilateral	95	1.24±0.78	1.0(1.0)	0.0	3.0	Z=0.81 p=0.419
	Bilateral	59	1.34±0.78	1.0(1.0)	0.0	3.0	
T1	Unilateral	95	2.35±1.00	2.0(1.0)	1.0	6.0	Z=6.12 p<0.001
	Bilateral	59	3.66±1.24	3.0(2.0)	2.0	6.0	
T2	Unilateral	95	1.84±0.88	2.0(1.0)	0.0	5.0	Z=5.57 p<0.001
	Bilateral	59	2.93±1.24	3.0(2.0)	1.0	6.0	
T3	Unilateral	95	1.53±0.73	1.0(1.0)	0.0	3.0	Z=4.60 p<0.001
	Bilateral	59	2.25±1.04	2.0(1.0)	0.0	5.0	

T0, before fracture; T1, after fracture and before treatment; T2, 1 week after treatment; T3, 1 month after treatment. Z, Mann-Whitney test.

**Table 4:** Comparison of SBQ score changes during intervals between groups.

Score changes	Group	N	Mean ± SD	Interquartile range	Min	Max	p value
T1 - T0	Unilateral	95	1.11±0.86	1.0(2.0)	0.0	3.0	Z=6.43 p<0.001
	Bilateral	59	2.32±1.07	2.0(1.0)	0.0	5.0	
T2 - T0	Unilateral	95	0.60±0.76	0.0(1.0)	0.0	3.0	Z=5.76 p<0.001
	Bilateral	59	1.59±1.07	2.0(1.0)	0.0	4.0	
T3 - T0	Unilateral	95	0.28±0.50	0.0(1.0)	0.0	2.0	Z=5.45 p<0.001
	Bilateral	59	0.92±0.79	1.0(1.0)	-1.0	3.0	
T2 - T1	Unilateral	95	-0.51±0.62	0.0(1.0)	-2.0	0.0	Z=1.82 p=0.069
	Bilateral	59	-0.73±0.74	-1.0(1.0)	-2.0	0.0	
T3 - T1	Unilateral	95	-0.82±0.70	-1.0(1.0)	-3.0	0.0	Z=4.22 p<0.001
	Bilateral	59	-1.41±0.87	-1.0(1.0)	-3.0	0.0	
T3 - T2	Unilateral	95	-0.32±0.53	0.0(1.0)	-2.0	0.0	Z=2.90 p=0.004
	Bilateral	59	-0.68±0.82	0.0(1.0)	-3.0	0.0	

T0, before fracture; T1, after fracture and before treatment; T2, 1 week after treatment; T3, 1 month after treatment. Z, Mann-Whitney test.

## Data Availability

The datasets generated during and/or analysed during the current study are available from the Correspondence on reasonable request.
